# Stakeholder driven development of a multi-criteria decision analysis tool for purchasing off-patent pharmaceuticals in Kuwait

**DOI:** 10.1186/s40545-019-0171-4

**Published:** 2019-04-16

**Authors:** Ali Hadi Abdullah, Anke-Peggy Holtorf, Maryam Al-Hussaini, Jacinthe Lemay, Maryam Alowayesh, Zoltán Kaló

**Affiliations:** 1 Kuwait Pharmaceutical Association, Kuwait City, Kuwait; 2 Health Outcomes Strategies GmbH, Colmarerstrasse 58, 4055 Basel, CH Switzerland; 3 Department of Pharmaceutical Sciences, College of Health Sciences, Public Authority for Applied Education and Training PAAET, Kuwait City, Kuwait; 4 Department of Pharmacology and Applied Therapeutics, Faculty of Pharmacy, Kuwait University, Kuwait City, Kuwait; 5 Department of Pharmacy Practice, Faculty of Pharmacy, Kuwait University, Kuwait City, Kuwait; 6 Syreon Research Institute, Budapest, Hungary; 7 Department of Health Policy and Health Economics, Eötvös Loránd University, Budapest, Hungary

**Keywords:** Kuwait, Pharmaceutical purchasing, MCDA, Multi-source pharmaceuticals, Pharmaceutical policy, Simple multi attribute rating technique, SMART

## Abstract

**Background:**

In Kuwait, the government is planning to improve the specifications for purchase of medicine and to improve the tendering system intending to slow the growth of the expenditure for medicine and to improve the sustainability of the healthcare system. Multiple Criteria Decision Analysis (MCDA) is a method which can help to assess multiple and sometimes conflicting criteria in the evaluation of the available alternatives. The objective of this initiative was to develop collaboratively a MCDA tool which is locally relevant, and which could be used to improve the use of performance indicators in the purchasing of off-patent pharmaceuticals.

**Methods:**

Nineteen leading experts representing a broad range of pharmaceutical policy stakeholders elaborated a locally adapted MCDA format by following a 7-step process for criteria selection, scoring, ranking and weighting.

**Results:**

The most important criterion was the price measured as savings versus the originator product with a weight of 35% in the final decision and a full score with a 60% price reduction. In addition, eight criteria were considered important for assessing the product performance in the Kuwaiti healthcare system: ‘equivalence with the reference product’ (weight of 16.2%), ‘stability and drug formulation’ (13.5%), ‘quality assurance’ (11.2%), ‘reliability of drug supply’ (8.8%), ‘macroeconomic benefit’ (5.5%), ‘real world outcomes (clinical and economic)’ (4.2%), ‘pharmacovigilance’ (3.3%), and ‘added value services related to the product’ (2.3%).

**Conclusions:**

A MCDA model was successfully adapted to the Kuwait decision context by a group of Kuwaiti pharmacists from a broad range of institutions. The participants agreed with the approach and considered it suitable to improve the transparency and consistency of decision making for off-patent pharmaceuticals in Kuwait. A pilot implementation project was proposed.

## Introduction

Strengthening medicine and medical supplies is among the key priorities of the national health plan set out by the Ministry of Health in Kuwait [1]. The current lack of an explicit national medicine policy has been identified by the ‘Country Cooperation Strategy for WHO and Kuwait’ as an area for policy improvement [1]. Kuwait is a wealthy country (GDP/capita US$ 69,900 est. 2017 [2]) in which the government provides many public services including good quality health care and education. Kuwait’s Public Authority for Civil Information estimates the country’s total population to be 4.6 Million for 2018, with immigrants (expatriates) accounting for more than 69.7% [3]. Healthcare services are provided to citizens and immigrants in Kuwait which are covered by public funding or an insurance scheme. The Ministry of Health through the Department of Medicines and Medical Equipment is managing the medicines and medical equipment. The Department has established sound registration, licensing, and quality assurance programs. Purchasing of pharmaceuticals happens through tenders conducted by the Central Medical Stores (CMS). Like in other GCC (Cooperation Council for the Arab States of the Gulf) countries, rational use of drugs is an issue, particularly its’ financial aspects. A national policy relating to generics or generic substitution has not yet been formulated [4]. The quality assurance system is operating well along with required laboratory analysis. Forward-looking, the government is planning to improve the specifications for purchase of common medicine and to improve the tendering system [1]. The overall objective is to slow the growth of the expenditure for medicines and thereby to improve the sustainability of the healthcare system.

The medicines most frequently used are off-patent pharmaceuticals, i.e. well established pharmaceuticals which have been on the market for a long time and therefore, are no longer protected by any patents. Many of these are multi-source products, meaning that multiple manufacturers are in competition for supplying these products [5]. There may be differences between the products delivered by different manufacturers which have critical impact on health outcomes, healthcare utilization, and cost [6–10]. It is therefore important to select the most efficient product and it has been suggested that factors related to the product (quality, bioequivalence, in some cases drug formulations), to the manufacturer (supply reliability, manufacturing quality), to the supporting evidence (clinical studies, pharmacovigilance, outcomes studies), or other value components (improved delivery, value added services) as well as cost should be considered in making the choice [11, 12].

Multiple Criteria Decision Analysis (MCDA) is a method which can help to assess multiple and sometimes conflicting criteria in the evaluation of the available alternatives [11]. Each criterion is scored separately and contributes with a predetermined weight, according to its importance, to the composite score reflecting the overall performance of the alternative. MCDA is being used widely across healthcare systems to inform decision making in healthcare, including benefit-risk assessment of medicines, formulary listing, purchasing, or reimbursement decisions [13–15]. MCDA has specifically been suggested as an evidence-based Health Technology Assessment (HTA) for evaluating off-patent pharmaceuticals in developing countries [11]. Examples for using MCDA in decision making for off-patent medicines in developing countries are emerging in several countries such as China, Thailand, or Egypt [16–19].

## Objective

The objective of this initiative was to collaborate among key decision makers in the access of medicines in Kuwait in order to develop a MCDA tool which is locally relevant, and which could be used to improve the use of performance indicators in the purchasing of off-patent pharmaceuticals.

### Methods

A 2-day workshop was conducted under patronage of the Kuwait Pharmacist Association in Kuwait with nineteen leading experts from the Central Medical Stores (3 persons), Pricing Department (1 person), Kuwait Drug and Food Control Administration (2 persons), hospital pharmacies (2 persons), non-governmental institutions (2 persons), primary healthcare (5 persons), the Public Authority for Applied Education and Training PAAET (1) and from the Faculty of Pharmacy at Kuwait University (3 persons). Moderated by 2 international health policy experts, a previously developed and validated MCDA model and process for local adaptation [11, 20] was used to guide the workshop participants through the local adaptation of the MCDA format using a structured seven-step process as depicted in.

**Fig. 1:**
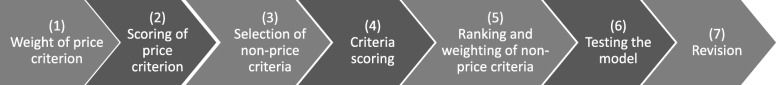
Seven-step process for developing the MCDA tool in a workshop with key decision makers in Kuwait

Fig. 1 The workshop started by defining the weight of the price criterion (Step 1) in the overall decision and the expected price reduction cut-off allowing for the maximum score in the price criterion (Step 2)

Subsequently, all other criteria relevant in the Kuwait decision process were defined starting from the basic decision criteria proposed by Brixner et al. [11] (Step 3). This involved a detailed discussion of each of the criteria and of the measures used for scoring each of the criteria (Step 4) as well as an anonymous voting with an Audience Response System for defining the Kuwaiti decision priorities and the relative importance of each of the criteria in the overall decision following the modified SMART (Simple Multi-Attribute Rating Technique) method [21] for ranking and swing weighting of the criteria (Step 5). In each voting, the result was computed by assessing the median value. The resulting model was tested using two test cases (Step 6). Finally, the participants had the opportunity to redefine or fine-tune some of the weights of the criteria based on the experience with the case studies (Step 7).

## Results

The discussion among the participants confirmed that currently, there is no uniform medicine policy applied to purchasing decision-making. Additional challenges were seen in the frequent issues with the supply chain management and the unclear implications of the GCC price harmonization. All participants agreed that acquisition cost is important in the purchasing decision, but that there are other important properties of off-patent products which should be accounted for. In Kuwait, there is a strong preference for the originator products [4]. However, due to budgetary constraints and increasing utilization, it is important to also include generic alternatives. The aim of a MCDA process was to identify those alternatives which offer the highest value in comparison to the originator product.

Step 1 (weight of price criterion): The first consensus to be reached was on the relative importance of the price criterion in the decision. The participants voted and the resulting median price weight in the overall evaluation was 42.5%. Consequently, the combined weight of all the non-price criteria would be 57.5% (100–42.5%).

Step 2 (scoring of price criterion): To be able to build a quantitative scoring function for the price criterion, the participants had to determine the cut-off point for the price. The cut-off-point in this model is the price reduction which allows for a full score for the pricing criterion. This cut-off was determined again by voting and the resulting median cut-off point was − 47.5%. This means that all alternatives with a price reduction versus the originator of − 47.5% or more would receive the full score in the evaluation.

Step 3 (selection of non-price criteria): The following criteria were identified to be most important in this comparison: *Equivalence* with the reference product, *Macroeconomic benefit*, *Pharmacovigilance*, *Quality assurance*, *Real world outcomes* (clinical and economic), *Reliability of drug supply*, *Stability and drug formulation*, and *Added value services* related to product. These were selected from a list of criteria which have been proposed previously on an international level as most relevant base criteria in the comparison and evaluation of off-patent pharmaceuticals in developing countries [11]. In addition, country of origin and package size were discussed as potentially important decision criteria. However, the participants were not able to form a consensus on objective and transparent performance measures for both criteria and therefore, both criteria were omitted from the further discussion. The participants concluded that country of origin is currently considered a substitute measure for quality, which in the new MCDA model is already addressed sufficiently by three of the eight base criteria (Equivalence with the reference product, Stability and drug formulation, Quality assurance). In relation to package size, the participants agreed that initially all comparisons should be made on equal units such as for example the defined daily dose. However, it was also noted that some forms of packaging may have implications for health outcomes (e.g. bulk product must be repacked by the pharmacist) or for efficiency (e.g. blisters with a standard number of doses may lead to product wastage), which potentially might also influence the purchasing decision and therefore, an inclusion to the model could be reconsidered at a later stage.

Step 4 (criteria measurement): For the majority of the criteria, the measurement scale (scoring) was adopted from those measures published previously [11, 22]. One exception was the product’s bioequivalence with the reference, where the proof of bioequivalence is the minimum requirement for registration in Kuwait and therefore, all products without such proof should be excluded from further consideration.

Step 5 (ranking and weighting of non-price criteria by ‘SMART and swing’ method): The results concerning the relative importance of the criteria and their weight in the overall scoring is summarized in Table 1 in the column ‘Initial Weights’.

**Table 1:** Results of the consensus workshop for the relative importance of the evaluation criteria and their weight in the final score for each option. *The initially determined weights were refined after the case study experience by reducing the weight of the price criterion. ** Health economic or health outcomes data

Criterion	Measures	Ranking	Initial Weights*	Final Weights*
Savings versus originator	Quantitative	Primary	42.5%	35.0%
Equivalence with the reference product	Qualitative	1	14.3%	16.2%
Stability and drug formulation	Qualitative	2	11.9%	13.5%
Quality assurance	Qualitative	3	9.9%	11.2%
Reliability of drug supply	Qualitative	4	7.8%	8.8%
Macroeconomic benefit	Qualitative	5	4.9%	5.5%
Real world outcomes**	Qualitative	6	3.7%	4.2%
Pharmacovigilance	Qualitative	7	2.9%	3.3%
Added value service related to product	Qualitative	8	2.1%	2.3%

Step 6 (testing of the model with previously designed case studies): Participants received the adapted model spreadsheet and assessed the performance of 4 alternative products for 2 different product categories. The conclusions from the testing were that (a) the weight of the pricing criterion was considered too high, and (b) the cut-off level of − 47.5% for the price reduction was not enough.

Step 7 (revision of price determinants): Both of price related model determinants were revised through a new voting process. The results were, that the new weight for the price criterion should be 35% and the cut-off point for maximum score should be changed to − 60%. The consequences for the new weight distribution in the overall MCDA model are shown in Table 1 in the column ‘Final Weights’.

**Fig. 2:**
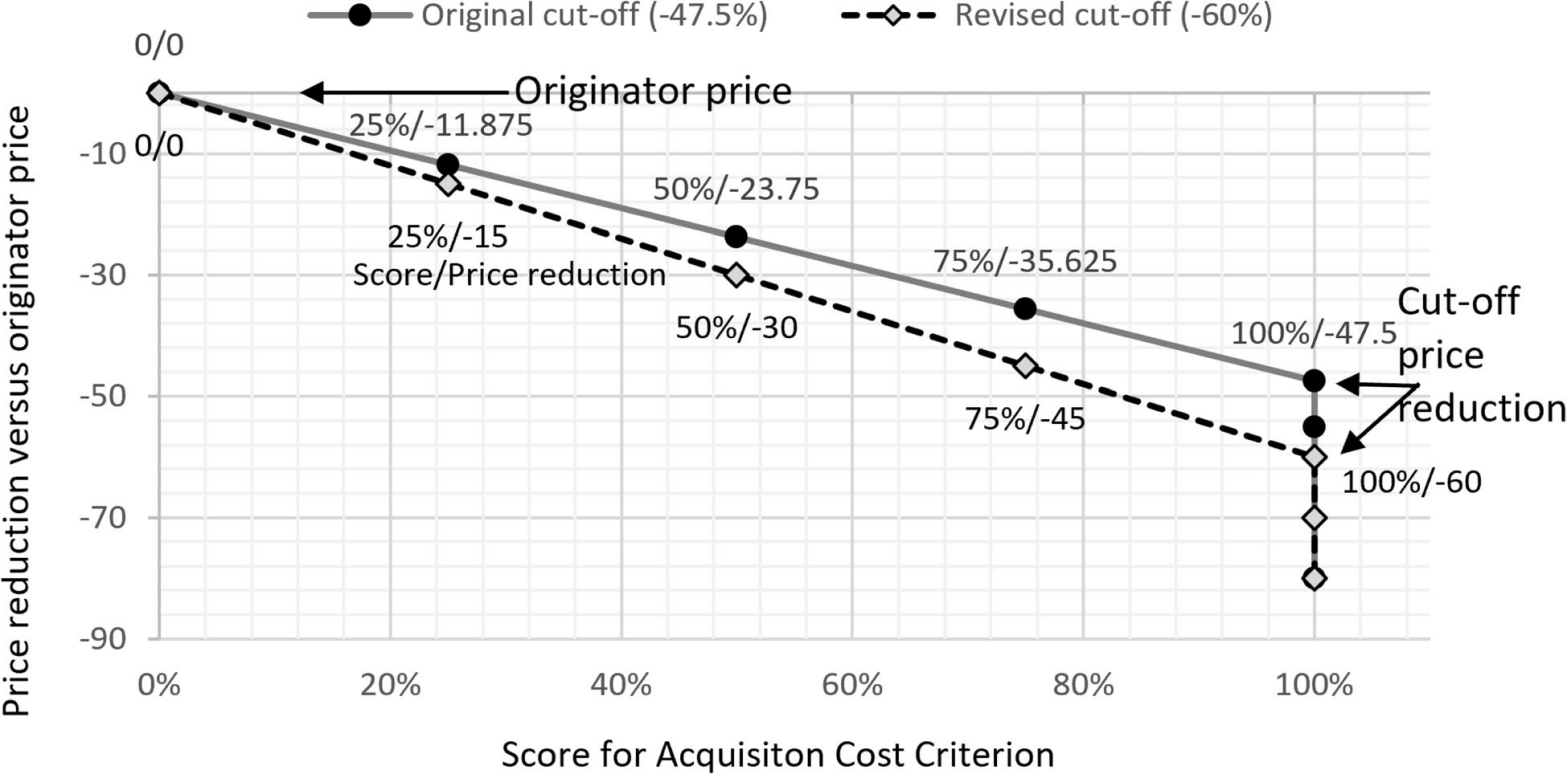
Graphic representation of the scoring for the price reduction of the alternatives versus the originator price. The original cut-off point determined in the workshop was − 47.5% meaning that all drugs offered at 52.5% of the originator price or below would receive a full score for the price criterion. The cut-off point was revised after the case study exercise to − 60%. Now, all prices at or below 40% of the originator price receive the full score. The scores between the originator price (Score = 0) and the full score follow a linear function

The impact of the decreased cut-off-point on the price scoring is shown in Fig. 2. The slope of the Score/Price-reduction function with the revised cut-off (− 60%) is steeper.

## Discussion

The final list of criteria selected for the Kuwait MCDA model is aligned with those which were previously suggested by an international expert group [11] and which were confirmed in other developing countries [22]. It is an essential principle of the MCDA methodology that the scoring in each of the criteria must be defined by transparent and objective measures. It was not possible to reach a consensus on such measures for two potential additional criteria (country of origin and package size) which were proposed in the Kuwait workshop and therefore, they were not adopted to the model for the time being. The MCDA model is a living instrument which can be revised when the priorities and needs in the healthcare system and policies change. Therefore, additional criteria can be included at a later stage once a consensus on the importance and the transparent measures for qualification is reached among the users of the instrument. For the majority of the measures used for scoring each of the criteria, the scales were adopted as proposed in the base model [11, 22]. Only for the equivalence criterion, a stricter rating scale was deemed more appropriate by the participants. Because the registration agency in Kuwait already applies very strict quality criteria for all registered pharmaceuticals, it was decided that all products without any proof of bioequivalence should be excluded immediately from further evaluation. The practical implication of this approach would be that products lacking the proof of bioequivalence can never be purchased for any potential use in Kuwait. The acceptance of this narrow interpretation of this decision criterion by all relevant stakeholders for purchasing of pharmaceuticals in Kuwait will have to be confirmed before implementing the decision tool on a broader level.

The ‘SMART and swing’ method [21] was successfully applied to determine the ranking and weighting of the criteria for Kuwait. The workshop resulted in a MCDA model which assesses products based on price reduction versus the originator product (with a weight of 35% in the final model) and 8 additional non-price criteria relating to product quality, manufacturer quality, services, and value aspects (with a combined weight of 65% in the final model). Among the non-price criteria, those relating to quality were deemed most important and have a combined impact of 40.9% on the product selection. Supply reliability of the manufacturer was also considered important and has an impact of 8.8% on the final product score. The impact of the four remaining criteria including macroeconomic benefit (local investment), real world outcomes, pharmacovigilance, and added value service related to product remains limited with a combined weight of 15.3%.

The importance of the price criterion had been discussed in the first step of the workshop. It should be noted that while price reduction is certainly a priority goal in the purchasing of off-patent pharmaceuticals, it should not be the only decision criterion. If the weight of the price criterion is too high, it would overrule any other decision criteria such as quality or availability. In the initial discussion and voting the price criterion received a weight of 42.5% in the overall decision, which leaves a combined weight of 57.2% to all other criteria.

An important first validation step is the testing of the resulting model where the participants can use pre-constructed realistic product examples to experience the implications of the model on their decisions. The 2 reference cases included an analgesic in the acute healthcare setting and an anti-hypertensive drug for chronic therapy. While the participants were generally satisfied with the use of the model, they expressed the concern that the price criterion might have been weighted too high in the initial model. An additional voting led to a reduction of the weight of the price criterion to 35%, leaving a combined weight of 65% to all other criteria.

In addition, the participants were concerned after testing the model that the relatively moderate cut-off point was not sufficiently differentiating between the product alternatives because already a price reduction of − 47.5% would qualify for a full price score. This may limit the incentive for manufacturers to offer the product at even lower prices. A second voting round was conducted for the two price related aspects, weight and cut-off point, and resulted in the final weight distribution as shown in Table 1 and a reduction in the cut-off point to − 60% as shown in Fig. 2.

Finally, all participants agreed that the resulting model seemed appropriate for the selection process in Kuwait and should be tested in a real-life pilot. A few areas were proposed as potential pilot applications, such as the selection of replacement products in the case of supply issues and drug shortages, or the selection of HIV, tuberculosis or oncology products for the hospital use. Realizing such a pilot application will require a second workshop with all stakeholders in the specific decision process and key decision makers in the Kuwait health policy to ensure full alignment around the process as well as development of a roadmap for a local implementation study. These stakeholders should be involved in the pilot itself and its evaluation to allow for full transparency, further improvement and finally, endorsement of the process in the Kuwait medicines decision context.

The purpose of the workshop with Kuwaiti pharmaceutical policy and purchasing stakeholders was to create a decision model tailored to the local processes and priorities. As a starting point, a set of internationally proposed criteria were suggested as base criteria [11]. These were refined to meet the needs of the participating stakeholders through a process of deliberation and consensus building. Similar workshops have been conducted previously in other countries, each resulting in a locally adapted set of decision criteria with different weighting and, to some degree, different scoring [17, 19, 20, 22, 23]. Local adaptation is essential to ensure that the model supports local pharmaceutical policy priorities. For example, in most countries, the decisions for off-patent pharmaceutical selection are mostly based on low price only, and therefore, the price comparator is the lowest price offering [17, 19, 20, 22]. However, in Kuwait there is currently a strong preference for the originator products. Therefore, it was very important in Kuwait to reflect this preference by using the originator price as comparator and by integrating criteria which ensure a high resemblance to the originator product in terms of quality, stability and reliability. To foster the adoption of the decision model to the local purchasing processes, it is essential that it reflects the local priorities and that the users recognize the resemblance of their previous decision priorities. If priorities and values of the local pharmaceutical policy change over time, the model can easily be adapted through regular revisions and adaptations using a similar consensus approach as presented in this report.

## Limitations

This exploratory workshop was conducted with a broad group of Kuwaiti pharmacy stakeholders. This group may not represent the final key stakeholders who would be involved in a concrete pilot project. Therefore, the current model may have to be challenged and revised in a second workshop once the concrete pilot application is identified. At that stage, additional stakeholders including the users/providers of the products and health policy decision makers should also be involved in order to maximize the face value of the model. In addition, not all measurement scales may prove to be practical in the everyday decision process. The feasibility will also have to be confirmed in the pilot phase, which should be followed by another revision of the model based on the experiences from the use in the real-life situation.

## Conclusions

A MCDA model was successfully adapted to the Kuwait decision context by a group of Kuwaiti pharmacists from a broad range of institutions. The participants agreed with the approach and considered it suitable to improve the transparency and consistency of decision making for off-patent pharmaceuticals in Kuwait. The final model included, in addition to price, eight important criteria for assessing the product performance in the Kuwaiti healthcare system.

## Abbreviations

CMS: Central Medical Stores
GCC: Cooperation Council for the Arab States of the Gulf
GDP: Gross Domestic Product
HIV: Human Immunodefinciency Virus
HTA: Health Technology Assessment
MCDA: Multiple Criteria Decision Analysis
PAAET: Public Authority for Applied Education and Training
SMART: Simple Multi-Attribute Rating Technique
WHO: World Health Organization
